# Effect of proton-conduction in electrolyte on electric efficiency of multi-stage solid oxide fuel cells

**DOI:** 10.1038/srep12640

**Published:** 2015-07-28

**Authors:** Yoshio Matsuzaki, Yuya Tachikawa, Takaaki Somekawa, Toru Hatae, Hiroshige Matsumoto, Shunsuke Taniguchi, Kazunari Sasaki

**Affiliations:** 1 Fundamental Technology Department, Tokyo Gas Co., Ltd., 1-7-7 Suehiro-cho, Tsurumi-ku, Yokohama City, Kanagawa 230-0045, Japan; 2 Next-generation Fuel Cell Research Center, Kyushu University, 744 Motooka, Nishi-ku, Fukuoka City, Fukuoka 819-0395, Japan; 3 International Institute for Carbon-Neutral Energy Research (WPI-I^2^CNER), Kyushu University, 744 Motooka, Nishi-ku, Fukuoka City, Fukuoka 819-0395, Japan; 4 Faculty of Engineering, Kyushu University, 744 Motooka, Nishi-ku, Fukuoka City, Fukuoka 819-0395, Japan; 5 International Research Center for Hydrogen Energy, Kyushu University, 744 Motooka, Nishi-ku, Fukuoka City, Fukuoka 819-0395, Japan

## Abstract

Solid oxide fuel cells (SOFCs) are promising electrochemical devices that enable the highest fuel-to-electricity conversion efficiencies under high operating temperatures. The concept of multi-stage electrochemical oxidation using SOFCs has been proposed and studied over the past several decades for further improving the electrical efficiency. However, the improvement is limited by fuel dilution downstream of the fuel flow. Therefore, evolved technologies are required to achieve considerably higher electrical efficiencies. Here we present an innovative concept for a critically-high fuel-to-electricity conversion efficiency of up to 85% based on the lower heating value (LHV), in which a high-temperature multi-stage electrochemical oxidation is combined with a proton-conducting solid electrolyte. Switching a solid electrolyte material from a conventional oxide-ion conducting material to a proton-conducting material under the high-temperature multi-stage electrochemical oxidation mechanism has proven to be highly advantageous for the electrical efficiency. The DC efficiency of 85% (LHV) corresponds to a net AC efficiency of approximately 76% (LHV), where the net AC efficiency refers to the transmission-end AC efficiency. This evolved concept will yield a considerably higher efficiency with a much smaller generation capacity than the state-of-the-art several tens-of-MW-class most advanced combined cycle (MACC).

To further enhance the electrical efficiency of solid oxide fuel cells (SOFCs), which enable the highest fuel-to-electricity conversion efficiencies under high operating temperatures^1^,^2^,^3^,^4^,^5^, it is necessary to increase the fuel utilization ratio (*Uf*) which refers to the quantity ratio of consumed fuel to supplied fuel. However, a high *Uf* (for example 90% or more) involves a considerable risk of running out of fuel at some cells in a stack and causing the oxidation of Ni in the Ni-YSZ (Yttria-Stabilized Zirconia) cermet typically used as the anode material, resulting in irreversible degradation. The primary reason for running out of fuel at some cells under a high *Uf* is the non-uniformity of the fuel distribution in the fuel cell stack. The risk is sufficiently low when the *Uf* in a stack is controlled to be approximately 75% or less.

Solid oxide fuel cells with a multi-stage electrochemical oxidation mechanism have been studied and developed over the past couple of decades to increase electrical efficiencies^6^,^7^,^8^,^9^. In the case of multi-stage electrochemical oxidation, the fuel supplied to an earlier stack placed upstream of the fuel flow is partially consumed through electrochemical oxidation in the stack and successively supplied to a latter stack placed downstream (Fig. 1). Then, the unutilized fuel from the earlier stack is delivered to the subsequent stack with zero waste, which results in increasing the *Uf* in the system while maintaining suppressed *Uf* in the respective stacks. Thus even with a *Uf* of the respective stacks of 75% or less, the multi-stage electrochemical oxidation technique enables entire system to operate at a *Uf* of 90% or more^8^. SOFCs with high electrical efficiencies of 68% (LHV, gross DC of the stack) and 60% (LHV, net AC of the system) have been reported^10^,^11^. The stack is known to have a two-stage electrochemical oxidation mechanism. Although this mechanism is effective, it has significant limitations due to the large dilution of the fuel. For further improvement in efficiencies with the multi-stage mechanism, “UltraFuelCell” has been proposed by U.S. Department of Energy (DOE), which combines the multi-stage SOFC with a gas turbine, and has a power generation capacity of 4 MW^8^.

SOFCs have oxide-ion conducting metal-oxide such as stabilized zirconia as electrolyte materials^2^, and they typically operate at temperatures higher than around 973.15 K. To reduce the operating temperature for improving the long-term stability, it is necessary to reduce the thickness of the electrolyte or to develop new electrolyte materials that have sufficiently high ionic conductivities even at the reduced temperatures^12^,^13^,^14^,^15^. As a kind of candidate materials for the new electrolyte, proton-conducting solid electrolytes have been studied^16^,^17^,^18^,^19^,^20^. Recently several studies have reported promising SOFC performances with proton-conducting electrolytes^21^,^22^.

In this work, we investigate an evolved concept for a critically-high fuel-to-electricity conversion efficiency, in which a high-temperature multi-stage electrochemical oxidation is combined with a proton-conducting solid electrolyte. Based on the most basic design of the multistage configuration, i.e., two-stage configuration, we have successfully shown the important findings and promising results on the electrical efficiency; we have demonstrated that simply switching a solid electrolyte material from a conventional oxide-ion conducting material to a proton-conducting material under the high-temperature multi-stage electrochemical oxidation mechanism results in highly advantageous for the electrical efficiency.

## Results

### A parametric study based on a symbolic analysis for the evolved concept

**Figure 1:**
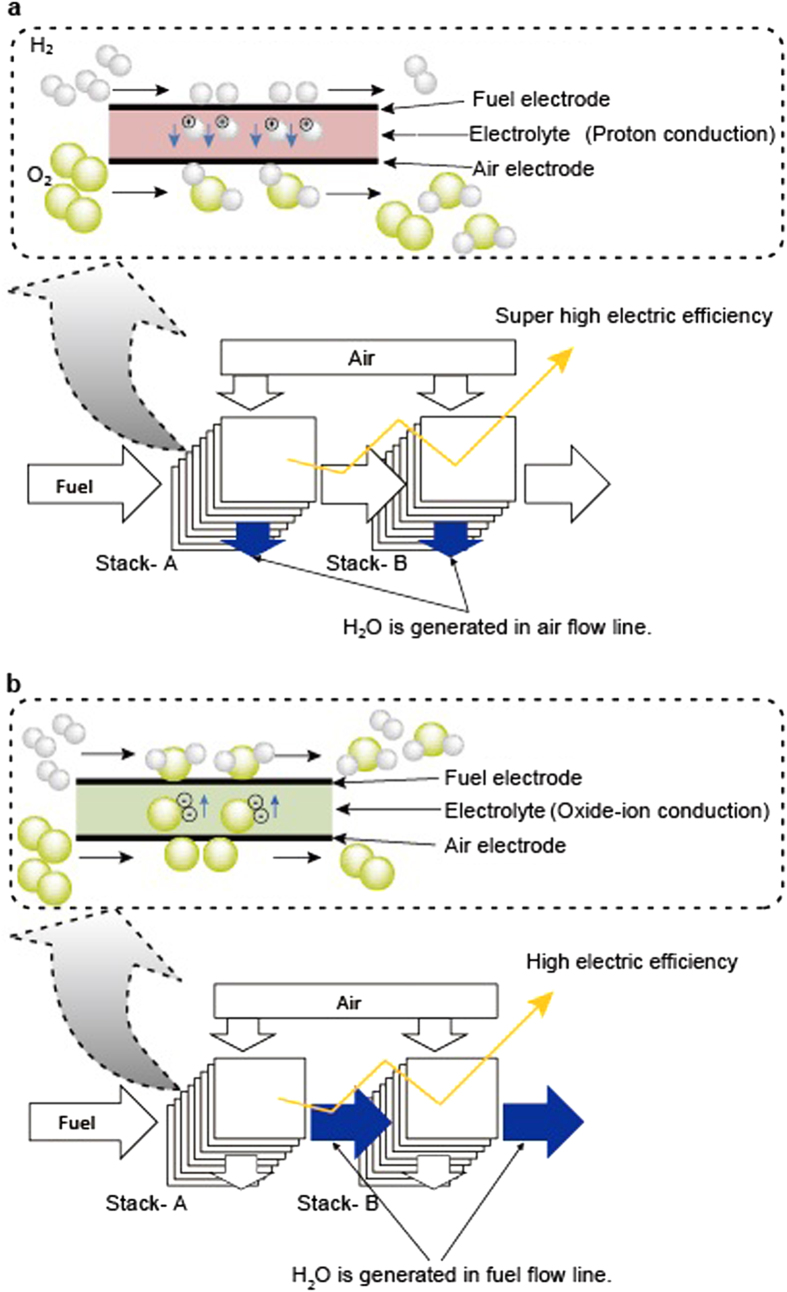
Schematic of the evolved concept presented in this work compared with the conventional two-stage electrochemical oxidation. (**a**) Evolved concept: high-temperature multi-stage electrochemical oxidation with proton-conducting oxide as an electrolyte (given as a two-stage example). The inserted magnification shows the mechanism of H_2_O production at the air electrode side. (**b**) Conventional multi-stage method with an oxide-ion conducting oxide as an electrolyte (given as a two-stage example). The inserted magnification shows the mechanism of H_2_O production at the fuel electrode side.

A schematic illustration of the evolved concept that we propose in this work, including the combination of high-temperature multi-stage electrochemical oxidation and a proton-conducting solid electrolyte, is shown in Fig. 1a, and it is compared with a conventional oxide-ion conducting electrolyte as shown in Fig. 1b. The inserted magnifications in Fig. 1a,b show the mechanism of steam generation at the air and fuel electrode sides, respectively. Because there is no steam generation in principle at the fuel electrode and no dilution of fuel by the generated steam, especially downstream, the combination of the high-temperature multi-stage electrochemical oxidation and the proton-conducting solid electrolyte is expected to enable outstandingly high electric efficiencies (Fig. 1a).

Therefore, we have investigated the capability of the combination for critically-high electric efficiencies by a parametric study based on a symbolic analysis, the method of which is described in the section of “Methods”. The most promising results obtained through the symbolic analysis were further evaluated by a chemical process simulator, Aspen Plus, based on a numerical analysis, the method of which is also described in the section of “Methods”, to test the feasibility of the critically high-efficient power generation. The area specific resistance (*ASR*) of the respective stacks is assumed to be 0.383 ohm cm^2^ at a sufficiently low *Uf* and at a temperature of 1,000 K. As described in the section of “Methods”, we defined the *ASR* of a single stack in individual use at a sufficiently low *Uf* as *ASR*_O_. The *ASR*_O_ value of 0.383 ohm cm^2^ is considered to be feasible for SOFC stacks^2^,^23^. Through the symbolic analysis, the electrical efficiency of a single stack in individual use with the *ASR*_O_ value is estimated to be 61% (LHV, gross DC) at a *Uf* of 75%, which corresponds to a net AC efficiency of 55% (LHV).

In the case of two-stage electrochemical oxidation, the *Uf* in stack- A placed upstream of the fuel, *Uf*_A_, and the *Uf* in stack- B placed downstream, *Uf*_B_, are expressed as functions of *Uf*_T_ and *r*, where *Uf*_T_ is the *Uf* in the entire system and *r* is the ratio of the fuel consumption in stack- B to stack- A. If the current density is equal in both stacks, the *r* value equals to the ratio of the active electrode area in stack- B to stack- A. In order to simplify the discussion, a fuel is assumed to be hydrogen temporarily. When the fuel is supplied to the inlet of stack- A at a rate of *Q* [mol sec^−1^], unutilized fuel will be supplied to the inlet of stack- B at a rate of 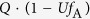, and then will be used in stack- B at a rate of 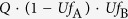. A total consumption rate of the fuel is the sum of the consumption rates in stacks- A and –B, 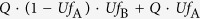, which should be divided by *Q* to give *Uf*_T_.
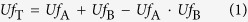


In addition, *r* is defined as consumption ratio of fuel in stack- B to that in stack- A, so that the following equation is derived.
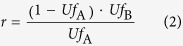


By solving the simultaneous equations (1) and (2), *Uf*_A_ and *Uf*_B_ can be obtained as functions of *r* and *Uf*_T_, which are expressed as equations (3) and (4), respectively. Equations (3) and (4) hold validity also for the case of the other fuels on the basis of Faraday’s law.
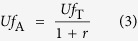

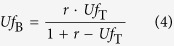


**Figure 2:**
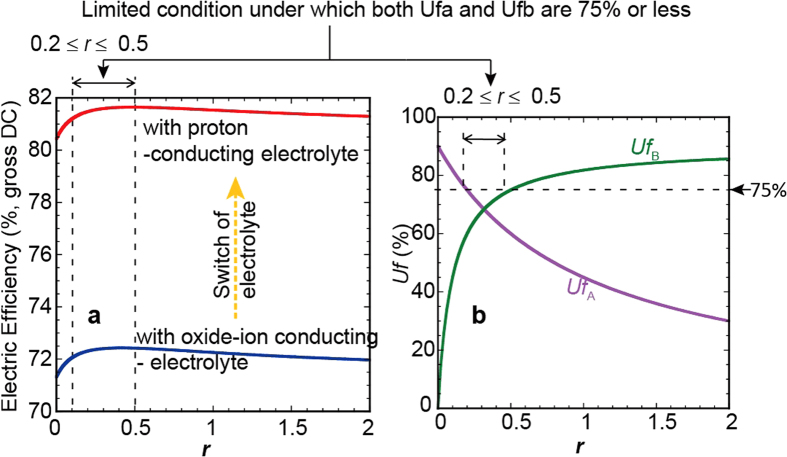
Electrical efficiencies with different types of charge-carrier (ion) in electrolytes in the case of the two-stage electrochemical oxidation, and the *Uf* of each stack. (**a**) The electrical efficiencies with a proton-conducting electrolyte and with an oxide-ion conducting electrolyte. (**b**) *Uf*_A_ and *Uf*_B_ as functions of *r*. Within the limited range of 0.2 ≤ *r* ≤ 0.5, both *Uf*_A_ and *Uf*_B_ have values of less than or equal to 75% simultaneously with a *Uf*_T_ of 90%.

As described in the section of “Methods”, the electrical efficiency also can be expressed as a function of *r* and *Uf*_T._ At a *Uf*_T_ of 90%, the electrical efficiency with methane as a fuel and the *Uf* in each stack were obtained as functions of *r* for the case of two-stage electrochemical oxidation through symbolic analysis (Fig. 2), where the partial pressure of H_2_O in air was assumed to be 2%. A remarkable increase in the electrical efficiency was observed simply by switching the type of charge-carrier (ion) in the electrolyte (Fig. 2a). This result indicates that the development of proton-conducting solid electrolyte with a high protonic transport number is important for realizing a critically-high efficiency.

The maximum electrical efficiency in the case of a proton-conducting electrolyte was found to be as high as 81.6% (LHV, gross DC) at an *r* of 0.5. It has become clear that there is a specific range of *r*, 0.2 ≤ *r* ≤ 0.5, in which it is possible to control *Uf*_A_ and *Uf*_B_ simultaneously to 75% or less even with a *Uf*_T_ of 90% (Fig. 2b). Thus, the electric efficiency is found to have outstandingly high values in the case of a proton-conducting electrolyte without a *Uf* of higher than 75% for each stack if the *r* value is adequately selected.

**Figure 3:**
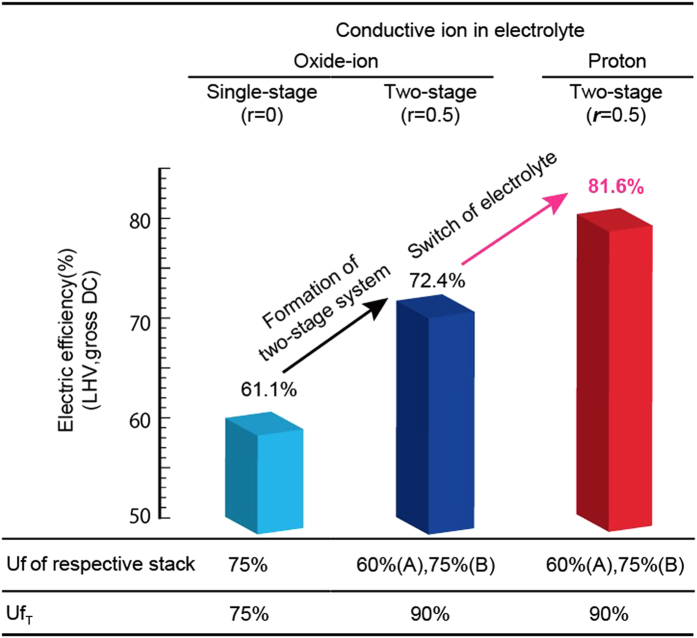
Main results from the symbolic analysis. The electrical efficiencies, the *Uf* of each stack, and *Uf*_T_ are listed for comparisons between the single- and two-stage electrochemical oxidation and between the oxide-ion conducting electrolyte and proton-conducting electrolyte. Configuration of the two-stage system and applying the proton-conducting electrolyte to the configuration enhanced the electrical efficiency from 61.1% to 81.6% (LHV, gross DC).

The efficiency and *Uf* at *r* = 0.5 are compared with the efficiency and *Uf* of single- and two-stage electrochemical oxidations with an oxide-ion conducting electrolyte (Fig. 3). *Uf*_A_ and *Uf*_B_ are calculated to be 60% and 75%, respectively, at a *Uf*_T_ of 90%. There are two types of mechanisms for reaching a critically-high efficiency; one is by the two-stage configuration, and the other is by the application of a proton-conducting electrolyte to the two-stage configuration (Fig. 3). Therefore, the symbolic analysis suggested that the evolved concept causes conventional SOFC stacks with electrical efficiencies of 61.1% (LHV, gross DC) in individual use to have significantly higher electrical efficiency of 81.6% (LHV, gross DC), even within a *Uf* range of 75% or less in each respective stack.

### Feasibility of the super-efficient power generation

**Figure 4:**
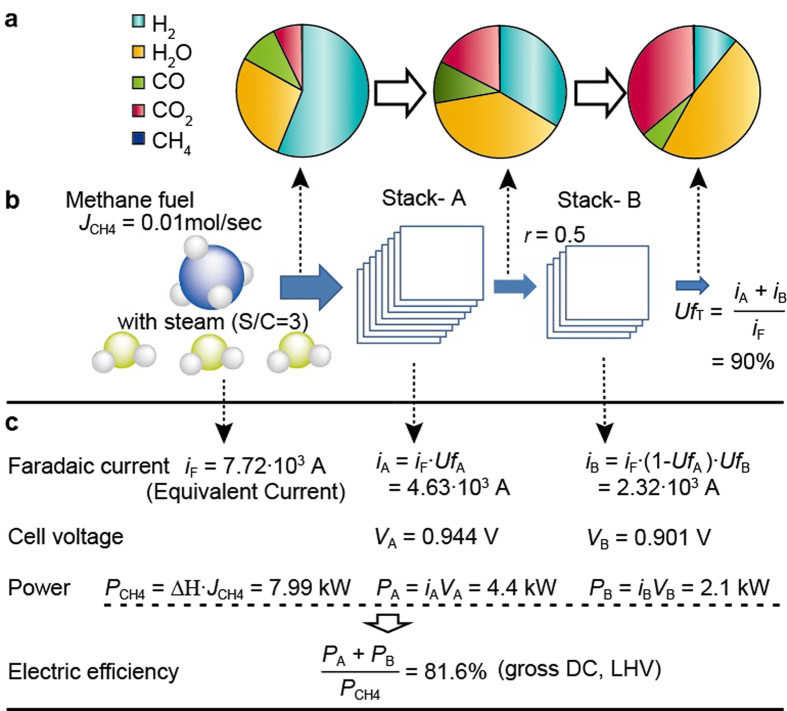
Schematic representations of the conditions and results of the numerical experiment with the equilibrium fuel compositions calculated by the symbolic analysis. (**a**) Equilibrium compositions at the inlet and outlet of the stacks. (**b**) Schematics of the two-stage electrochemical oxidation of methane with the conditions of the numerical simulation. (**c**) Faradaic current, cell voltage, output electrical power, and electrical efficiency when methane is supplied at the rate of 0.01 mol sec^−1^.

To study the feasibility of the critically-high efficiency predicted by the symbolic analysis, we have conducted a numerical experiment (chemical process simulation) which simulates supplying methane as a fuel to stack- A at the rate of 0.01 mol sec^−1^ mixed with H_2_O under the condition of *r* = 0.5 and *Uf*_T_ = 90% (Fig. 4b). The *r* value of 0.5 gave the maximum electrical efficiency in Fig. 2a. This condition corresponds to the *Uf*_A_ and *Uf*_B_ of 60% and 75%, respectively. The numerical experiment was conducted in AspenPlus Ver. 7.3. The molar flow rate of methane corresponds to 7.99 kW (LHV) of calorific value, and the faradaic current and cell voltage dictate the electrical output power of each stack. The electrical output powers of stacks- A and -B were calculated to be 4.4 and 2.1 kW, respectively at a current density of 0.25 A cm^−2^ (Fig. 4c). The electrical efficiency is given as a function of these output powers and the calorific value. The electrical efficiency thus calculated through the numerical simulation was 81.6% (LHV, gross DC) which corresponds well with the value predicted by the symbolic analysis. In this manner the critically-high efficiency has been reproduced by the numerical simulation.

Fuel cells with proton-conducting electrolytes cannot use CO as a fuel directly^2^. Therefore, the equilibrium composition of the fuel especially at the downstream stack (stack- B) has a significant impact on the feasibility of a critically-high efficiency. CH_4_ reacts with H_2_O to produce H_2_, CO, and CO_2_ at a stage prior to the inlet of stack- A, and H_2_ is consumed in stacks- A and -B in series. The concentration of H_2_O in the downstream equilibrium compositions, shown in pie charts in Fig. 4a, are considerably smaller than with the oxide-ion conducting electrolyte (see supplementary material - Figure S1 online). However, the H_2_O concentration is sufficient for a reaction with CO (i.e., the water-gas shift reaction) if H_2_ is consumed, which results in supporting H_2_ resupply. Consequently, in the case of the proton-conducting electrolyte under the conditions assumed in this study, CO fills an adequate role as an indirect fuel, supporting the feasibility of critically-high efficiency.

## Discussion

If the stacks have measurable gas leakage or ion-leakage, an upper limit of the *Uf* of not only the individual stacks but also the entire system should be considered. In such a case, optimizing the parameters with a constant *Uf*_T_ as shown in Fig. 2a will be applicable to designing the multi-stage configuration. Figure 2 indicates that the electrical efficiency has a maximum at *r* = 0.5 with the upper limit of *Uf*_T_ of 90%. Under this condition, *Uf*_A_ and *Uf*_B_ are calculated by the equations (3) and (4) to be 60% and 75%, respectively.

On the other hands, if there are no needs for the upper limit of the *Uf*_T_, only upper limits of the *Uf*_A_ and *Uf*_B_ should be considered. Without the upper limit of the *Uf*_T_, the *Uf*_A_ could be increased up to the assumed upper limit of 75% with accompanying a rise in the electrical efficiency. The electrical efficiency increased from 81.6% to 84.6% (LHV, gross DC) with increasing the *Uf*_A_ from 60% to 75% at a fixed *Uf*_B_ of 75% (Table 1). The DC efficiency of 84.6% corresponds to a net AC efficiency of approximately 76.1% (LHV). Equations (1) and (2) indicate that the *Uf*_T_ and *r* also change with increasing *Uf*_A_ with a fixed value of *Uf*_B_ (Table 1).

We have investigated the combination of proton-conducting electrolyte and the multi-stage SOFCs by using the most basic configuration, i.e., two-stage configuration, and have successfully shown the valuable findings and promising results on the electrical efficiency. Based on the findings, additional technologies would be applicable for further improving the electrical efficiency, for example such as three- or higher numbers- stage configurations, and/or multi-stage supplies of fresh fuels to the downstream stacks mixed with the exhausted fuels from the upstream stacks. In those technologies, there are lots of variable parameters to be investigated further to achieve higher efficiencies.

In this work, the proton-conducting electrolyte was assumed to have a protonic transport number of 1. In practice, however, the transport number depends on the material and temperature. Several approaches have been studied for increasing the protonic transport number and open-circuit voltage (OCV) to close to 1 and the theoretical value, respectively. For example, decreasing the operating temperature is effective^24^,^25^. Another approach is blocking oxide-ion by using Pd film which is reported to show OCV close to theoretical value and high performances^26^,^27^. Novel perovskite-type material with pure proton conductivity and chemical stability has also been reported^28^. These approaches are expected to produce an increased potential for realizing the evolved concept.

At high electrical efficiencies and high-temperatures, thermally self-sustaining operation is an important issue to consider^29^,^30^, and the smallest possible power-generating capacity of an SOFC system is determined mainly by a capability of a thermally self-sustaining operation. Here, a thermally self-sustaining operation entails maintaining the operation temperature of the system only by the heat generated from the system itself. In the case of the proton-conducting electrolyte, the electrochemical oxidation of hydrogen (exothermic reaction) will occur at the air electrode side. Therefore, under the thermally self-sustaining operation with sufficient margin, the stack with proton-conducting electrolyte will be cooled by air effectively, resulting in the BOP design similar to the case of the conventional stacks (based on oxide-ion conducting electrolytes). While the capability of a thermally self-sustaining operation and the smallest possible power-generating capacity of an SOFC system largely depend on the electrical efficiencies, operating temperatures, and structures of the stacks and systems, relatively much smaller power-generating capacities even with high electrical efficiencies are expected to be acceptable to SOFC systems (see supplementary material – Figure S2 online).

In summary, our study showed that simply switching the type of charge-carrier (ion) in the electrolyte from oxide-ion to proton yields remarkable advantages for the electrical efficiency under a high temperature multi-stage electrochemical oxidation mechanism. In this simple manner, an existing stack would be able to be used nearly as is except for changing a material in the layers with a thickness of approximately 10 μm consisting of electrolyte sandwiched with active electrodes. This result indicates that proton-conducting solid electrolytes with high protonic transport numbers as well as with long-term stability will become key materials for the technical innovation in the energy field. The efficiency is considerably higher than that of the state-of-the-art several tens-of-MW-class MACC^31^, and would consist with a critically smaller electrical output capacity (see supplementary material – Figure S2 online).

## Methods

### Methods related to both the symbolic analysis and the numerical experiment

Twelve parameters are considered for the two-stage electrochemical oxidation: such as *Uf*_T_, *Uf*_A_, *Uf*_B_, *r,* the temperature, the steam to carbon ratio (*S/C*), the current densities of stacks- A and -B, *ASR*_O_, the air utilization ratio (*Uair*) of stack- A, the *Uair* of stack- B, and partial pressure of H_2_O in air (see supplementary material – Figure S5-1 online). The following parameters are given assumed constants: the current density of stacks- A and -B is 0.25Acm^−2^, *S/C* is 3, the temperature is 1,000 K, the *Uair* of stacks- A and -B is 30%, the partial pressure of H_2_O in air is 2%, and the *ASR*_O_ is 0.383 ohm cm^2^.

The *ASR*_O_ is defined as the *ASR* of the single-stack in individual use at sufficiently low *Uf* and *Uair*. The *ASR*_O_ value is assumed to be 0.383 ohm cm^2^. As generally observed, the *ASR* estimated from the current-voltage characteristics has a *Uf* dependence. *ASR* used in this study, which is defined as ∆*V* (the difference between the cell voltage calculated by equation (5) and OCV) divided by the current density, also had a *Uf* dependence (Supplementary material Figure S5-2).

Considering the manufacturing cost of the stacks, stacks- A and -B were assumed to be stacked as an apparent single-stack with internal manifolds which enable the series connection of fuel supply as the two-stage configuration. Therefore, the current density was assumed to be equal for both stacks- A and -B.

All the parameters are used as variable parameters, assumed constants, or dependent parameters in accordance with the aim of the analysis (supplementary material – Table S5 online). In Fig. 2, *Uf*_T_ was assumed to be 90%, *Uf*_A_ and *Uf*_B_ were dependent parameters, *r* was used as a variable to optimize the dependent parameters under the upper limit of *Uf*_T_. In Fig. 3 (two-stage configuration), and Fig. 4, *Uf*_T_, *Uf*_A_, *Uf*_B_, and *r* were assumed to be 90%, 60%, 75%, 0.5, respectively, which gave the maximum efficiency under the upper limit of *Uf*_T_ of 90%. In Table 1, *Uf*_T_ and *r* were dependent parameters, *Uf*_B_ was fixed at 75% (assumed upper limit), *Uf*_A_ was used as a variable to calculate the *Uf*_A_ dependence of the electrical efficiency without the upper limit of *Uf*_T._

The cell voltage, *V*_cell_, in each stack is approximately calculated by using the *EMF*s of the inlet and outlet of the cell corresponding to the fuel compositions, the area specific resistance (*ASR*_O_), and the current density (*i*) as described in equation (5).
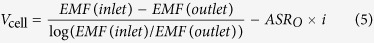


The first term on right side of this equation is the logarithmic mean of the *EMF*s at the inlet and outlet of the cell. The *EMF* is determined by the oxygen partial pressures, *P*O_2_, for the case of the oxide-ion conducting electrolyte, and the hydrogen partial pressures, *P*H_2_, for the case of the proton-conducting electrolyte, by equations (6) and (7), respectively, where R is the molar gas constant, and F is the Faraday constant. The effectiveness of the approximate expression, equation (5), has been confirmed by measurements of the dependence of the cell voltage on the *Uf* (see supplementary material – Figure S3 online).
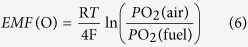

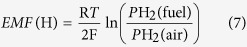


For confirmation, the geometric and arithmetic means of *EMF*s were compared with the logarithmic mean for the approximate calculations of the cell voltage. The two types of means yielded nearly the same voltage as that given by the logarithmic mean used for equation (5) (see supplementary material – Figure S4 online); thus, these averaging methods are also appropriate in addition to the logarithmic mean for the approximate calculation of the cell voltage.

### Parametric study based on a symbolic analysis

MAPLE 17 was used for the parametric study based on the symbolic analysis. The equilibrium composition of fuel including *P*O_2_ and *P*H_2_ at each stage used in equations (6) and (7) is determined by three equilibrium reactions (8)-(10) and the elemental mole fractions of C, H, and O.
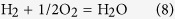

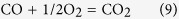

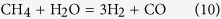


The Gibbs free energies of the reactions were taken from the JANAF table for the symbolic analysis using MAPLE 17. The element mole fractions at the inlet and outlet of the respective stacks were determined as a function of the independent variables, *Uf*_T_ and *r*.

### Calculation of the electrical efficiency in the case of the symbolic analysis

Electrical efficiency (gross DC), *η*_dc_ (symbolic), was calculated by using equation (11) as a function of *V*_ave_, *Uf*_T,_ and dH, where dH is the standard enthalpy change of the oxidation reaction of CH_4_ at 298.15 K and *V*_ave_ is a weighted average of the cell voltages of stack- A (*V*_A_) and stack- B (*V*_B_) as expressed by equation (12) (see supplementary material – Figure S6 online). The conversion loss from DC power to AC power was assumed to be 10%, so the net AC electrical efficiency, *η*_ac_, was calculated by equation (13). In the case of 700 W-class combined heat and power systems with fuel cells for residential use, the power loss from DC to AC was reported to be 100 W (from 800 to 700 W), which corresponds to a loss of 12.5%^32^. In the case of 1.5 kW-class systems, the efficiency loss from DC to AC was reported to be 8 percentage point (from 68% to 60%), which corresponds to a loss of 11.8%^10^,^11^. The evolved concept we discussed in this study was based on the premise of a larger generating capacity of several- kW or more. Because the power loss ratio tends to decrease with an increase in the generating capacity, we assumed the power loss ratio of 10% in equation (13).
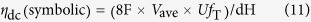

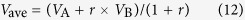

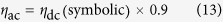


### Numerical experiment (process simulation)

A process simulation based on the numerical analysis of the total system process, which includes not only two-stage SOFC stacks but also auxiliary devices such as heat exchangers, a fuel reformer, a fuel combustor, and a vaporizer, and a heat recovery unit, the so-called balance of plant (BOP), was numerically modeled at the steady state by using AspenPlus Ver. 7.3 (see supplementary material – Figure S7 online). Equilibrium composition of the fuel at each stage was calculated by using a thermodynamics database from AIChE, which contains the thermodynamic functions and equilibrium parameters of the vapour-phase molecules appearing in this study. After reforming, fuel was immediately supplied to stack- A followed by stack- B, and the fuel was finally subject to complete combustion at the fuel combustor. The flow lines of air supply to the stacks were in parallel.

Methane, air, and water were supplied to the inlet of the system at 298.15 K. The flow rate of methane was set to 1.0 × 10^−2^ mol sec^−1^ mixed with water at a flow rate of 3.0 × 10^−2^ mol sec^−1^. The system operating conditions were defined as follows: *S/C* was 3, the air utilization ratio of each stack was 30%, and the excess air ratio in the fuel combustor was 1.05. Several auxiliary devices were thermally integrated in the system. The thermal energies needed for the reforming reaction, the vaporization of water, and the pre-heating of air and fuel, were covered by the heat generated at the stacks and the combustion chamber under thermal management using several heat exchangers (see supplementary material – Figure S7 online).

### Calculation of electrical efficiency in the case of the numerical analysis

The faradic currents in stacks- A and -B are given by equations (14) and (15) when methane fuel is supplied to stack- A at *M*_CH4_ mol sec^−1^.
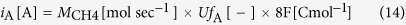

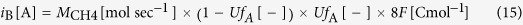


The electrical output powers *P*_A_ and *P*_B_ in stacks- A and -B were calculated by equations (16) and (17). Using the electrical output powers and enthalpy of the supplied methane with a flow rate of *M*_CH4_, the electrical efficiency (gross DC), *η*_dc_ (numeric), was calculated by equation (18)
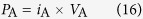

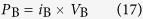

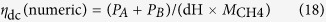


## Additional Information

**How to cite this article**: Matsuzaki, Y. *et al.* Effect of proton-conduction in electrolyte on electric efficiency of multi-stage solid oxide fuel cells. *Sci. Rep.* **5**, 12640; doi: 10.1038/srep12640 (2015).

## Supplementary Material

Supplementary Information

## Figures and Tables

**Table 1:** Electrical efficiency (*η*_e_), *Uf*_T_, and *r* as a function of *Uf*_A._

**Given parameters**		**Results**		
***Uf***_**A**_	***Uf***_**B**_	***r***	***Uf***_**T**_	***η***_**e**_
60%	75%	0.500	90.0%	81.4%
65%	75%	0.404	91.3%	82.7%
70%	75%	0.321	92.5%	83.7%
75%	75%	0.250	93.8%	84.6%

The electrical efficiency increased from 81.6% to 84.6% (LHV, gross DC) when the *Uf*_A_ increased from 60% to 75% with a fixed *Uf*_B_ of 75%
