# Supplementary material for: Effect of proton-conduction in electrolyte on electric efficiency of multi-stage solid oxide fuel cells
Source: Sci Rep. 2015 Jul 28;5:12640. doi: 10.1038/srep12640 (PMC4517511; doi:10.1038/srep12640)
Supplement: Supplementary Information [file srep12640-s1.pdf]

for submission to *Scientific Reports*

Effect of proton-conduction in electrolyte on electric efficiency of multi-stage solid oxide fuel cells

Yoshio Matsuzaki[1,2,*], Yuya Tachikawa[3], Takaaki Somekawa[1,4], Toru Hatae[1], Hiroshige Matsumoto[3], Shunsuke Taniguchi[5] & Kazunari Sasaki[2,3,4,5]

[1]Fundamental Technology Department, Tokyo Gas Co., Ltd., 1-7-7 Suehiro-cho, Tsurumi-ku, Yokohama City, Kanagawa 230-0045, Japan.

[2]Next-generation Fuel Cell Research Center, Kyushu University, 744 Motooka, Nishi-ku, Fukuoka City, Fukuoka 819-0395, Japan.

[3]International Institute for Carbon-Neutral Energy Research (WPI-I^{2}CNER), Kyushu University, 744 Motooka, Nishi-ku, Fukuoka City, Fukuoka 819-0395, Japan.

[4]Faculty of Engineering, Kyushu University, 744 Motooka, Nishi-ku, Fukuoka City, Fukuoka 819-0395, Japan.

[5]International Research Center for Hydrogen Energy, Kyushu University, 744 Motooka, Nishi-ku, Fukuoka City, Fukuoka 819-0395, Japan.

*Correspondence and requests for materials should be addressed to Y.M. (matuzaki@tokyo-gas.co.jp).

Supplementary material

S1. Equilibrium composition at each stage
in the case of the oxide-ion conducting electrolyte

The fuel at the inlet and outlet of stack- B in the two-stage electrochemical oxidation with the oxide-ion conducting electrolyte has been found to be highly diluted with steam compared with the proton-conducting electrolyte shown in Fig. 4a, which could significantly limit increases in the electrical efficiency.

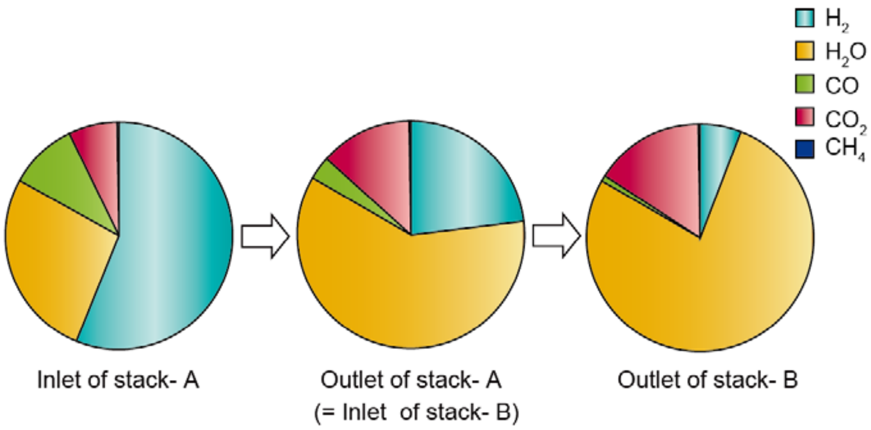

Figure S1. Equilibrium compositions with two-stage electrochemical oxidation in the case of the oxide-ion conducting electrolyte.

S2. Comparisons of the electrical efficiencies with a variety of power generation capacities

The evolved concept proposed in this study will consist with a considerably higher efficiency and critically smaller capacity than the state-of-the-art several tens-of-MW-class MACC. The possible minimum power generation capacity of the SOFC system is determined mainly by the capability of the thermally self-sustaining operation. SOFC systems with electrical efficiency of 46% and 60% (LHV, net AC) have been reported to consist with electrical output capacities of 0.7 and 1.5 kW, respectively[3,5]. The smallest possible power generation capacity is assumed to increase in proportion to the volume of high-temperature space, and the difference in heat loss between the systems is proportional to both the difference in the surface area of the high-temperature space and the difference in the input energy of the fuel after deducting the electrical output power considering thermal balance. Simple estimation from these values under these assumptions results in an output electrical capacity of 4.0 kW for a thermally self-sustaining operation with a critically-high efficiency of 74% (LHV, net AC). However, the capability of a thermally self-sustaining operation depends largely on the operating temperatures and structures of stacks and systems. Therefore, the minimum scale necessary for the super-efficient system with a thermally self-sustaining operation should be defined based on the details of a specific system design.

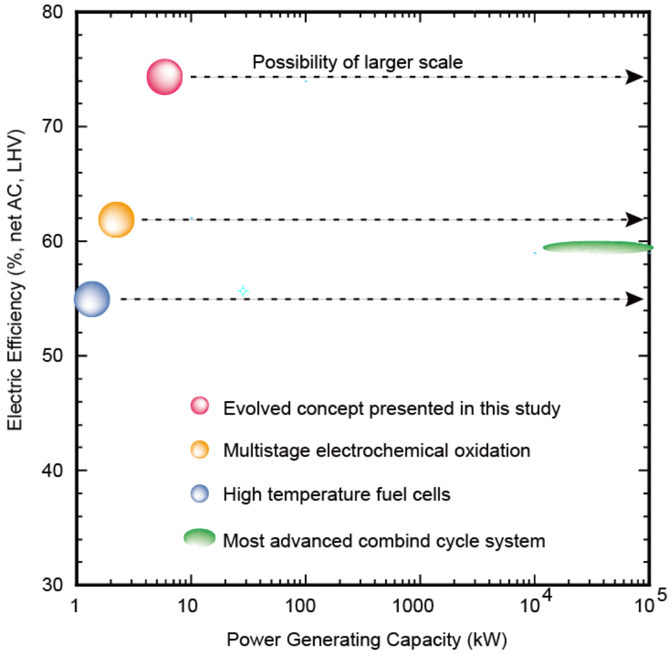

Figure S2. Transmission-end AC electrical efficiency versus power generation capacity.

S3. logarithmic mean of the *EMF*s used for approximate calculation of the cell voltage

The slope of the logarithmic mean of the *EMF*s at the inlet and outlet of the cell corresponded well with that of the experimental data of the cell voltage. The gap between the logarithmic mean of the *EMF*s and the cell voltage is explained as resulting from the voltage drop due to the internal resistance of the cell as described in Eq. (3).

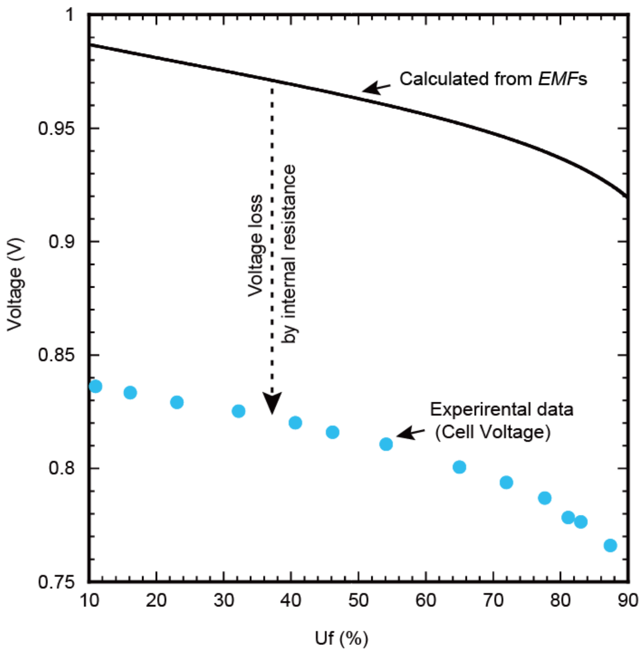

Figure S3. Experimental data of the cell voltage and logarithmic mean of the *EMF*s.

S4. Geometric and arithmetic means of *EMF*s compared with the logarithmic mean

The two types of means yielded nearly the same voltage as the logarithmic mean; thus, these averaging methods are also appropriate in addition to the logarithmic mean for the approximate calculation of the cell voltage.

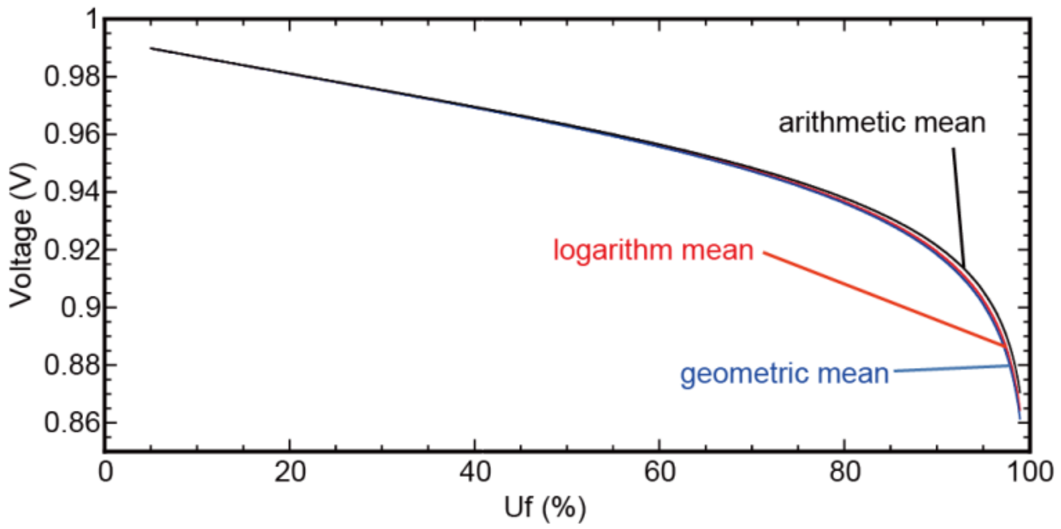

Figure S4. Geometric and arithmetic means of *EMF*s compared with the logarithmic mean for the approximate calculations of the dependence of the cell voltage on the *Uf*.

S5. Parameters and assumptions considered in this study

The twelve main parameters to be considered for the two-stage electrochemical oxidation, which are described on rectangular blue backgrounds, are Uf_T, Uf_A, Uf_B, *r*, the temperature, *S/C*, the current densities of stacks- A and -B, *ASR*, the *Uair* of stacks-A and -B, and the partial pressure of H_2O in air.

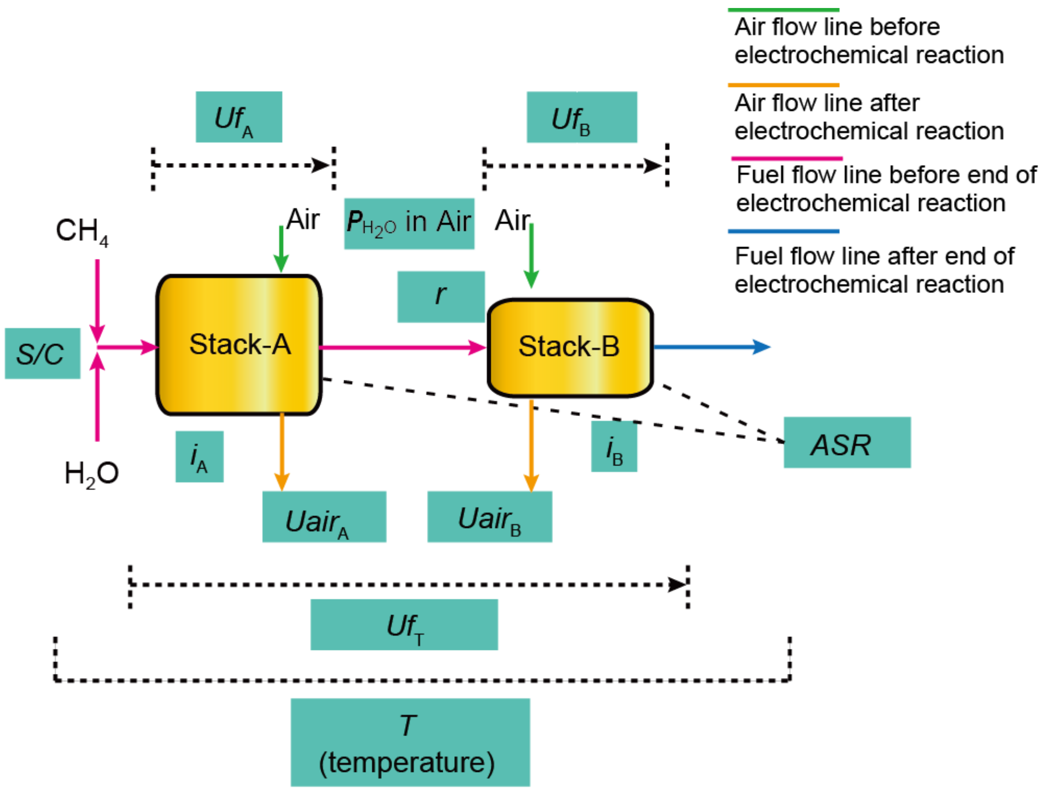

Figure S5-1. Main parameters for the two-stage electrochemical oxidation.

Table S5. List of parameters and assumptions used in this study

1) Uf_T
   - assumed to be 90% in Fig. 2 for optimization with upper limit of Uf_T
   - assumed to be 90% in Fig. 3 (two-stage), and Fig. 4
   - function of Uf_A and Uf_B in Table 1 for calculation without upper limit of Uf_T
2) Uf_A
   - function of Uf_T and *r* in Fig. 2 for optimization with upper limit of Uf_T
   - assumed to be 60% in Fig. 3 (two-stage), and Fig. 4
   - variable in Table 1 for calculation without upper limit of Uf_T
3) Uf_B
   - function of Uf_T and *r* in Fig. 2 for optimization with upper limit of Uf_T
   - assumed to be 75% in Fig. 3 (two-stage), and Fig. 4)
   - assumed to be 75% in Table 1 for calculation without upper limit of Uf_T (upper limit of individual stack was assumed to be 75%)
4) *r*
   - variable in Fig. 2 for optimization with upper limit of Uf_T
   - assumed to be 0.5 in Fig. 3 (two-stage), and Fig. 4
   - function of Uf_A and Uf_B in Table 1 for calculation without upper limit of Uf_T
5) Temperature
   - assumed to be 1000 K
6) *S/C*
   - assumed to be 3
7) Current Density of Stack-A
   - assumed to be 0.25 Acm^{-2} (same as Stack- B)
8) Current Density of Stack-B
   - assumed to be 0.25 Acm^{-2} (same as Stack- A)
9) ASR_O
   - assumed to be 0.383 ohm cm^2
10) *Uair* of Stack-A
   - assumed to be 30% (same as Stack- B)
11) *Uair* of Stack-B
   - assumed to be 30% (same as Stack- A)
12) Partial Pressure of H_2O in air.
   - assumed to be 2%

The *ASR* of the single-stack in individual use at sufficiently low *Uf* and *Uair* was defined as ASR_O, and it was assumed to be 0.383 ohm cm^2. The *ASR* defined as ΔV (the difference between the cell voltage and OCV) divided by the current density, showed a *Uf* dependence. The *Uf* dependence of *ASR* with oxide-ion conducting electrolyte is shown as an example.

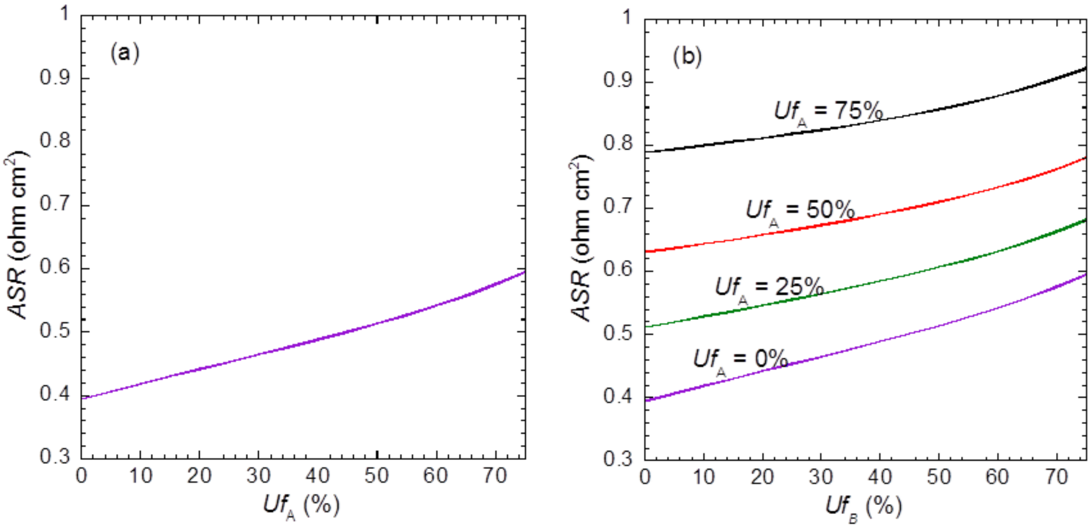

Figure S5-2. *Uf* dependence of *ASR* with the two-stage design and oxide-ion conducting electrolyte at *Uair*=30%. (a) and (b) indicates Stack- A and Stack- B, respectively.

S6. Dependence of cell voltages on the *r* value

Both the cell voltages of stacks- A and -B increase with *r* because the *EMF* at the outlet of stack- A, which is equal to that at the inlet of stack- B, increases with *r* due to decrease in Uf_A. The weighted average has a maximum because the effect of increase of the cell voltages is dominant at relatively small *r* values and the effect of the increase in the weight of the lower voltage, V_B, is dominant at large *r* values.

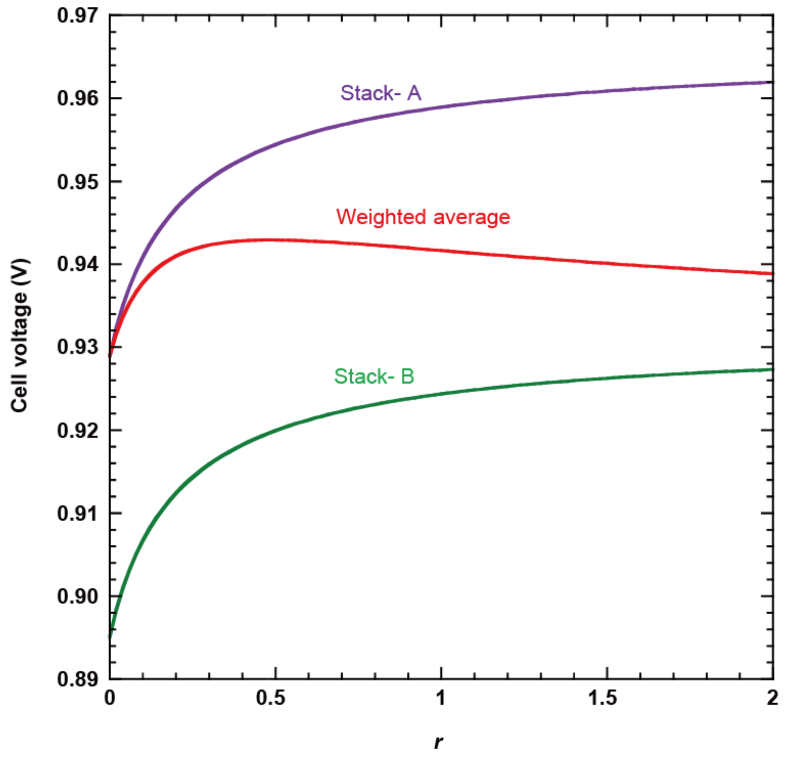

Figure S6. Cell voltages of stacks- A, -B with proton-conducting electrolyte and the weighted average of the voltages as a function of *r*.

S7. System model for the system process analysis

The flow lines of fuel and air to the stacks are in series and in parallel, respectively. The input temperatures of air, fuel, and water were set to 298.15 K. The heat generation in the system is equal to the difference between the enthalpy change by the oxidation of CH_4 at the stacks and in the combustion chamber and the total amount of electrical output power (P_A+P_B). The generated heat was primarily used for the reformer, the vaporizer, and the pre-heating of the air and fuel. The thermal output of the system was assumed to be obtained by hot water through the heat recovery device.

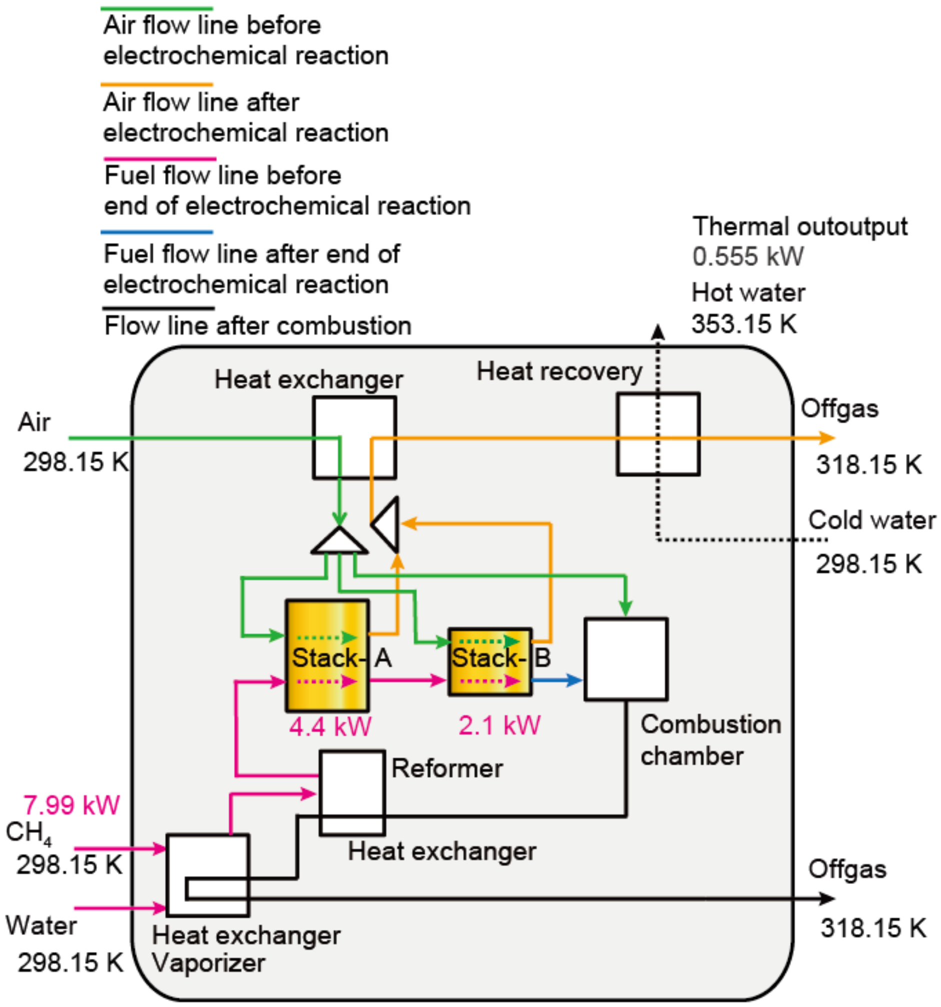

Figure S7. Schematic representation of a system model used for system process analysis.
